# Protection against Dengue Virus Infection in Mice by Administration of Antibodies against Modified Nonstructural Protein 1

**DOI:** 10.1371/journal.pone.0092495

**Published:** 2014-03-21

**Authors:** Shu-Wen Wan, Yi-Tien Lu, Chia-Hui Huang, Chiou-Feng Lin, Robert Anderson, Hsiao-Sheng Liu, Trai-Ming Yeh, Yu-Ting Yen, Betty A. Wu-Hsieh, Yee-Shin Lin

**Affiliations:** 1 Center of Infectious Disease and Signaling Research, National Cheng Kung University, Tainan, Taiwan; 2 Department of Microbiology and Immunology, College of Medicine, National Cheng Kung University, Tainan, Taiwan; 3 Institute of Clinical Medicine, College of Medicine, National Cheng Kung University, Tainan, Taiwan; 4 Department of Microbiology and Immunology, Dalhousie University, Halifax, Nova Scotia, Canada; 5 Department of Medical Laboratory Science and Biotechnology, College of Medicine, National Cheng Kung University, Tainan, Taiwan; 6 Graduate Institute of Immunology, College of Medicine, National Taiwan University, Taipei, Taiwan; The University of Chicago, United States of America

## Abstract

**Background:**

Infection with dengue virus (DENV) may cause life-threatening disease with thrombocytopenia and vascular leakage which are related to dysfunction of platelets and endothelial cells. We previously showed that antibodies (Abs) against DENV nonstructural protein 1 (NS1) cross-react with human platelets and endothelial cells, leading to functional disturbances. Based on sequence homology analysis, the C-terminal region of DENV NS1 protein contains cross-reactive epitopes. For safety in vaccine development, the cross-reactive epitopes of DENV NS1 protein should be deleted or modified.

**Methodology/Principal Findings:**

We tested the protective effects of Abs against full-length DENV NS1, NS1 lacking the C-terminal amino acids (a.a.) 271-352 (designated ΔC NS1), and chimeric DJ NS1 consisting of N-terminal DENV NS1 (a.a. 1-270) and C-terminal Japanese encephalitis virus NS1 (a.a. 271-352). The anti-ΔC NS1 and anti-DJ NS1 Abs showed a lower binding activity to endothelial cells and platelets than that of anti-DENV NS1 Abs. Passive immunization with anti-ΔC NS1 and anti-DJ NS1 Abs reduced DENV-induced prolonged mouse tail bleeding time. Treatment with anti-DENV NS1, anti-ΔC NS1 and anti-DJ NS1 Abs reduced local skin hemorrhage, controlled the viral load of DENV infection *in vivo*, synergized with complement to inhibit viral replication *in vitro*, as well as abolished DENV-induced macrophage infiltration to the site of skin inoculation. Moreover, active immunization with modified NS1 protein, but not with unmodified DENV NS1 protein, reduced DENV-induced prolonged bleeding time, local skin hemorrhage, and viral load.

**Conclusions/Significance:**

These results support the idea that modified NS1 proteins may represent an improved strategy for safe and effective vaccine development against DENV infection.

## Introduction

Dengue virus (DENV) belongs to the Flaviviridae family of enveloped, positive-strand RNA viruses, and is transmitted by *Aedes* mosquitoes. With increased international travel and climate change, the prevalence of dengue is spreading beyond its usual tropical and subtropical boundaries. Hence, dengue is becoming one of the most important health issues in the world. DENV infection causes variable clinical presentations ranging from mild dengue fever to severe dengue hemorrhagic fever (DHF) and dengue shock syndrome (DSS). The major clinical manifestations of DHF/DSS are thrombocytopenia and vascular leakage, although additional symptoms, such as liver damage, may occur [1]-[4].

So far, there is no vaccine or specific antiviral drug available. One of the obstacles is the lack of suitable animal models for human dengue disease. DENV can infect nonhuman primates but does not replicate well or cause substantial disease. For reasons of cost and convenience, mouse models have been used to test vaccine candidates prior to testing in nonhuman primates. Recent progress has been made in modeling certain aspects of human dengue disease in mice. Following intravenous, intraperitoneal, intracerebal or intradermal inoculation of DENV, mice show liver pathology, thrombocytopenia, neurological symptoms, or hemorrhage [5]. A dengue hemorrhage mouse model has been established which mimics the natural route of infection in humans. This model gives rise to severe thrombocytopenia, prolonged bleeding time, and increased numbers of circulating endothelial cells. TNF-α produced by monocytes and infiltrating macrophages enhances production of DENV-induced reactive nitrogen species and reactive oxygen species and also leads to endothelial cell damage [6]–[8]. Hence, the murine model can be used to test for many parameters of vaccine efficacy including hemorrhage which is a common clinical manifestation seen in DHF/DSS patients.

For dengue vaccine development, the envelope (E) and precursor membrane (prM) proteins are major antigens for inducing protective antibody (Ab) responses. However, Abs against E and prM are not only neutralizing but also enhancing which facilitate DENV infection through Ab-dependent enhancement (ADE) [9]–[15]. On the other hand, nonstructural protein 1 (NS1) is also an important target of Abs induced by DENV and is a candidate for vaccine development. As a nonstructural protein, NS1 can avoid the risk of ADE. Importantly, anti-NS1 Abs trigger complement-mediated lysis of DENV-infected cells [16]. Furthermore, several studies showed that active immunization with NS1 protein or NS1 DNA vaccine, as well as passive immunization with anti-NS1 Abs provided protection for mice from DENV challenge [16]–[20]. A downside to the use of NS1 as a vaccine, however, is that anti-NS1 Abs may cross-react with human coagulation factors, adhesion molecules or LYRIC (lysine-rich CEACAM1 co-isolated) on platelets, and endothelial cells [21]–[24].

We previously showed that anti-DENV NS1 Abs cross-react with platelets and inhibit platelet aggregation [25]. In addition, anti-DENV NS1 Abs can bind to endothelial cells and cause cell apoptosis or inflammatory activation [26], [27]. Based on proteomic and sequence homology analysis, the C-terminal region of DENV NS1 protein contains cross-reactive epitopes shared with several self-antigens [28]. Therefore, we deleted the C-terminus of DENV NS1 proteins from amino acids (a.a.) 271-352 to generate ΔC NS1 proteins. Anti-ΔC NS1 Abs showed a lower binding activity to human platelets and did not inhibit platelet aggregation. Moreover, DENV NS1, but not ΔC NS1, immunization caused prolonged bleeding time in mice [29]. In the present study, we tested the protection provided by Abs against modified NS1 proteins against DENV challenge. Besides ΔC NS1 protein, we generated a chimeric DJ NS1 protein, which consisted of N-terminal DENV NS1 (a.a. 1-270) and C-terminal Japanese encephalitis virus (JEV) NS1 (a.a. 271-352). The Abs against full-length DENV NS1, ΔC NS1 and DJ NS1 proteins were assayed for their pathogenic or protective effects both *in vitro* and *in vivo*.

## Materials and Methods

### Mice

C3H/HeN breeder mice were obtained from Charles River Breeding Laboratories. They were maintained on standard laboratory food and water in our medical college laboratory animal center. Their 8-week-old progeny were used for the generation of Abs and their 4-5-week-old progeny were used for protection studies. Housing, breeding, and experimental use of the animals were performed in strict accordance with the Experimental Animal Committee of National Cheng Kung University.

### Preparation of recombinant proteins and Abs

The cDNA of DENV2 NS1 (New Guinea C strain) was cloned into the pRSETb vector containing a His-tag. ΔC NS1 (deletion of a.a. 271-352) and DJ NS1 (a.a. 1-270 of DENV NS1 and a.a. 271-352 of JEV NS1) cDNA were cloned into the pET28a vector also containing a His-tag. The plasmids were introduced into *E. coli* BL21. The recombinant proteins were induced by 1 M isopropyl B-D-1-thiogalgactopyranoside (IPTG) (Calbiochem) and purified with Ni^2+^ columns. After purification, proteins were examined using 10% SDS-PAGE. Proteins from SDS-PAGE were excised and homogenized in Freund's adjuvant (Sigma-Aldrich) to intraperitoneally (i.p.) immunize mice five times at a dose of 25 μg. The first dose was administered in complete Freund's adjuvant (CFA) and the following four doses were given in incomplete Freund's adjuvant (IFA). Mouse sera were collected three days after the last immunization. The polyclonal Abs against DENV NS1, ΔC NS1 and DJ NS1 from hyperimmunized sera were purified with protein G agarose columns (Millipore) and recovered with HCl-glycine. The control IgG was eluted from a protein G column loaded with normal mouse sera. The preparations were subjected to testing for endotoxin contamination using a Limulus amebocyte lysate assay (Pyrotell, Associates of Cape Cod), the endotoxin concentrations of anti-DENV NS1, anti-ΔC NS1, anti-DJ NS1 and control IgG were all <0.03 EU/ml.

### Bleeding time

Bleeding time was performed by a 3-mm tail-tip transection. Blood droplets were collected on filter paper every 30 sec. Bleeding time was recorded when the blood spot was smaller than 0.1 mm in diameter [29], [30].

### Cell and virus cultures

Human microvascular endothelial cell line-1 (HMEC-1) was obtained from the Centers for Disease Control and Prevention (Atlanta) [31], and passaged in culture plates using endothelial cell growth medium M200 (Cascade Biologics) containing 2% FBS, 1 μg/ml hydrocortisone, 10 ng/ml epidermal growth factor, 3 ng/ml basic fibroblast growth factor, 10 μg/ml heparin, and antibiotics.

DENV2 (strain 16681) was propagated in C6/36 cells. Briefly, C6/36 monolayers were incubated with DENV at multiplicity of infection (MOI) of 0.01 at 28°C in 5% CO_2_ for 5 days. The cultured medium was harvested, and cell debris was removed by centrifugation at 900×*g* for 10 min. After further centrifugation at 16,000×*g* for 10 min, the virus supernatant was collected and stored at −70°C until use in experiments. Virus titer was determined by plaque assay using BHK-21 cells as described previously [32].

### Platelet preparation

Human whole blood (collected by the ethical approval from the Institutional Review Board of National Cheng Kung University Hospital, No. A-ER-102-123, with written informed consent obtained from healthy volunteers) containing the anticoagulant ACD (29.9 mM sodium citrate, 113.8 mM glucose, 72.6 mM sodium chloride and 2.9 mM citric acid, pH 6.4) was centrifuged at 1000×*g* for 10 min at room temperature. The upper layer as platelet-rich plasma was removed to a 15-ml tube and washed twice in EDTA/PBS. The washed platelets were suspended in Tyrode's solution (137 mM NaCl, 20 mM HEPES, 3.3 mM NaH_2_PO_4_, 2.7 mM KCl, 1 mg/ml BSA and 5.6 mM glucose, pH 7.4) at a concentration of 10^8^ platelets/ml [29].

### Flow cytometry

For platelet and endothelial cell binding assay, the washed platelets and endothelial cells were fixed with 1% formaldehyde at room temperature for 10 min and then washed with PBS. Control IgG, anti-DENV NS1, anti-ΔC NS1 and anti-DJ NS1 Abs were incubated with platelets or endothelial cells at 4°C for 1 h. After washing twice with PBS, the samples were incubated with 1 μl of 1 mg/ml Alexa-488-conjugated goat anti-mouse IgG (Invitrogen) at 4°C for 30 min. The binding activity was analyzed by flow cytometry (FACSCalibur; BD Biosciences) with excitation set at 488 nm. The percentage of positive cells was determined by comparison with the control IgG group.

### Animal protection model

Mice were injected i.p. with Abs (100 μg/mouse), followed one day later by intradermal (i.d.) inoculation with DENV (9×10^7^ pfu/mouse) at four sites on the upper back as previously described [6]. On days one and two post-infection, mice were further injected i.p. with Abs and were sacrificed at day 3 after inoculation.

Proteins from SDS-PAGE were excised and homogenized in Freund's adjuvant to i.p. immunize mice three times at a dose of 25 μg (once in CFA and then twice in IFA). Three days following the final immunization, mice were i.d. inoculated with DENV (9×10^7^ pfu/mouse) at four sites on the upper back and sacrificed at day 3 after inoculation.

### Histopathology

Mouse skin was fixed in 10% neutral-buffered formalin solution and then dehydrated in graded alcohol. The fixed tissue was embedded in paraffin and sliced into 4-μm-thick sections. The skin sections were mounted in regular glass slides and stained with hematoxylin and eosin.

### Immunohistochemistry staining

The skin sections were embedded in paraffin and sliced on slides. Using xylene and gradient alcohol (100%, 95%, 85%, 70% and 50%) to deparaffin, the sections incubated in 2N HCl solution for 20 min and then with 20 μg/ml proteinase K in TE buffer (50 mM Tris Base, 1 mM EDTA, 0.5% Triton X-100, pH 8.0) for another 20 min at room temperature. The sections were incubated with 3% H_2_O_2_ in water for 15 min to inhibit endogenous peroxidase activity and blocked by 10% BSA in PBS.

The primary and secondary Abs were adequately diluted in Ab diluents (Dako Corporation). Cells containing DENV antigen were detected with anti-DENV NS3 Abs (GeneTex) overnight at 4°C, followed by HRP conjugated goat anti-rabbit Abs at room temperature for 1 h (Jackson Immunoresearch Laboratories). The infiltrating macrophages were stained with anti-mouse F4/80 Abs (Serotec) overnight at 4°C, followed by biotin-labeled donkey anti-rat Abs (Jackson Immunoresearch Laboratories) at room temperature for 2 h. After washing with PBS, the sections were incubated with streptavidin (Dako Corporation) at room temperature for 15 min. The skin sections were developed using the AEC substrate kit (Vector Laboratories) and counterstained with hematoxylin. The sections were also analyzed using a TissueFAXS (TissueGnostics Vienna, Austria) image cytometer and quantified with the HistoQuest software (TissueGnostics) using an average of 15 fields of view. HistoQuest separated the Ab-mediated chromogen (AEC) stain and the counterstain. Results were displayed as dot plots with each dot representing a single cell in the tissue sample.

### Immunofluorescence staining

The skin tissues were frozen in liquid nitrogen and the cryosections were fixed in 4% formaldehyde for 3 min and acetone for 5 min at room temperature. The sections were then blocked with 10% BSA in PBS for 30 min at room temperature. Sections were stained with anti-NS3 and anti-CD31 Abs (BD Biosciences) in Ab diluents overnight at 4°C, followed by Alexa-488-conjugated donkey anti-rabbit Abs (Invitrogen) and Alexa-594-conjugated donkey anti-rat Abs (Invitrogen) at room temperature for 2 h. The nuclei of cells were stained with DAPI (Calbiochem). Fluorescence images were detected using an Olympus BX-51 microscope.

### Ab-dependent complement-mediated cytolytic assay

HMEC-1 cells were inoculated with DENV (MOI = 10) for 1 h at 37°C and then washed twice with medium. After centrifugation to remove residual virus in the culture supernatant, 5×10^3^ cells were seeded into each well of 96-well tissue-culture plates for further incubation in 5% CO_2_ in a 37°C incubator. At 72 h post-infection, DENV-infected HMEC-1 cells (about 45% E antigen-positive) were harvested and incubated with Abs (50 μg/ml) at 4°C for 1 h. After washing twice with PBS, cells were incubated with Low-Tox-M rabbit complement (1∶20 dilution, Cedarlane Laboratories Ltd) for 4 h in 5% CO_2_ in a 37°C incubator to facilitate complement-mediated cell lysis. Cytolysis was measured by the release of lactate dehydrogenase (LDH), a cytoplasmic enzyme, with a commercial kit (Cytotoxicity detection kit, Roche Diagnostics). The optimal density was determined using a microplate reader set to 490 nm.

### Effect of Abs and complement on DENV replication

To measure the effect of Abs and complement on virus production *in vitro*, HMEC-1 cells in 96-well plates were infected with DENV at an MOI of 10 for 1 h. Residual extracellular virus was then removed by washing three times with medium. After 6 h incubation, the medium was then replaced with medium containing Abs (50 μg/ml) and complement (1∶20). Cells were incubated for 72 h at 37°C in a 5% CO_2_ incubator. At 24 h intervals, culture supernatants were collected for determination of infectious virus titer by plaque assay.

### Statistics

Comparisons between various treatments were performed by unpaired *t*-test with GraphPad Prism version 5.0. Statistical significance was set at *P*<0.05.

## Results

### Absence of pathogenic effects of Abs against modified NS1 proteins lacking cross-reactive epitopes both *in vitro* and *in vivo*

**Figure 1:**
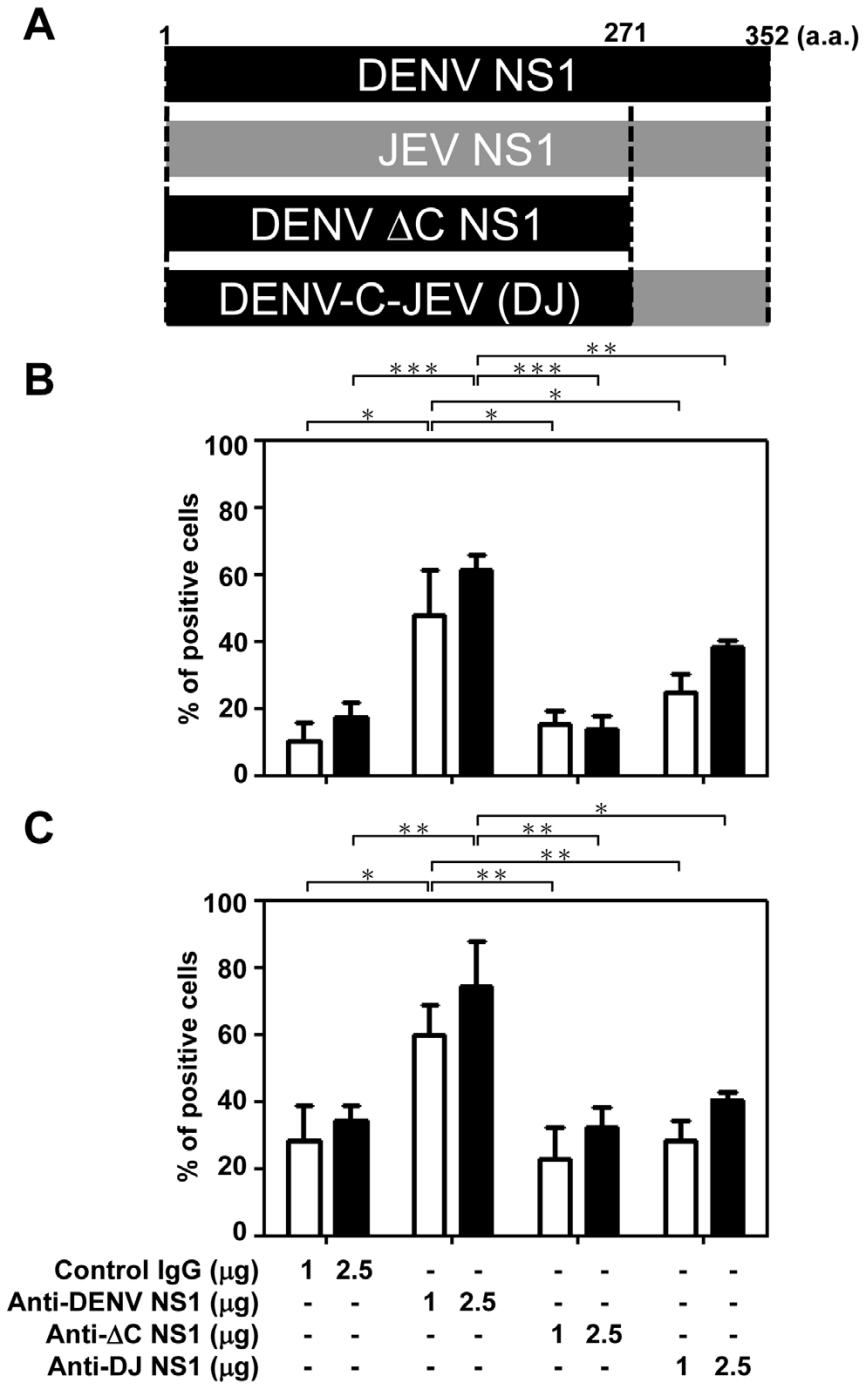
Anti-ΔC NS1 and anti-DJ NS1 Abs show lower binding activity to human endothelial cells and platelets than anti-full-length DENV NS1 Abs. (A) The C-terminal region of DENV NS1 protein from a.a. 271-352 was deleted to generate ΔC NS1 protein. The DJ NS1 protein consists of the N-terminus of DENV NS1 (a.a. 1-270) and the C-terminus of JEV NS1 (a.a. 271-352). Polyclonal Abs against DENV NS1, ΔC NS1, or DJ NS1 were generated in mice and purified on protein G columns. Formaldehyde-fixed human endothelial cells (B) and platelets (C) were incubated with control IgG, anti-DENV NS1, anti-ΔC NS1 or anti-DJ NS1 Abs, followed by Alexa 488-conjucated anti-mouse IgG and analyzed by flow cytometry. The averages of triplicate cultures are shown. * *P*<0.05, ** *P*<0.01, *** *P*<0.001.

**Figure 2:**
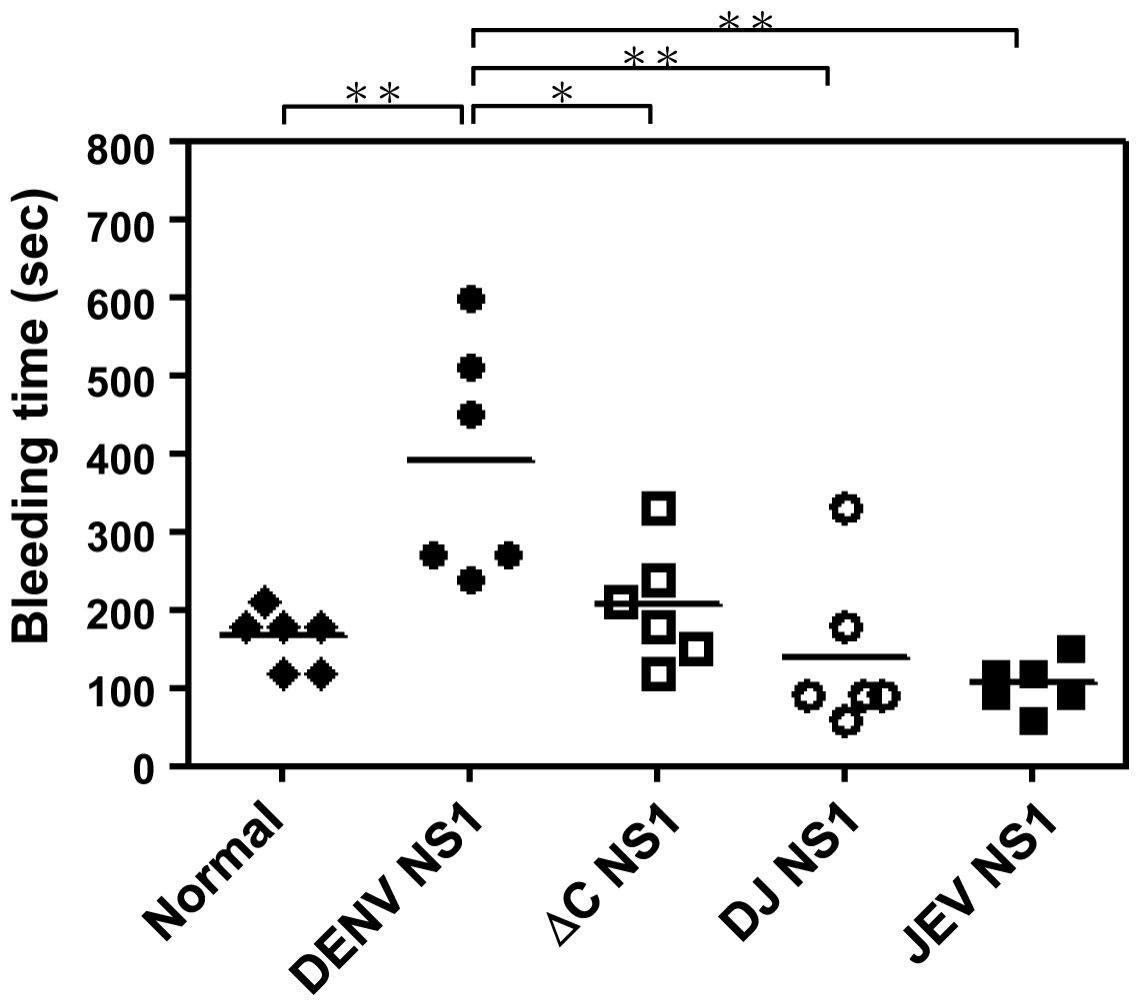
Effect of immunization with DENV NS1, JEV NS1, ΔC NS1 and DJ NS1 proteins on bleeding time in mice. C3H/HeN mice (n = 6/group) were intraperitoneally (i.p.) immunized five times with DENV NS1, JEV NS1, ΔC NS1 or DJ NS1 proteins. The mouse tail bleeding time was determined 3 days after the last immunization. Prolonged bleeding time was observed in DENV NS1-immunized mice, but not in ΔC NS1- and DJ NS1-immunized mice. * *P*<0.05, ** *P*<0.01.

We previously found that anti-DENV NS1 Abs cross-react with platelets and endothelial cells [25], [26] and the cross-reactive epitopes are located predominantly in the C-terminal region of the DENV NS1 protein [28], [33]. We therefore deleted the C-terminus of DENV NS1 protein from a.a. 271-352 to generate ΔC NS1 protein. We also generated a chimeric DJ NS1 protein, which consists of N-terminal DENV NS1 (a.a. 1-270) and C-terminal JEV NS1 (a.a. 271-352) (Figure 1A). The Abs against DENV NS1, ΔC NS1 or DJ NS1 proteins were purified from sera of immunized mice and tested for binding activity to human endothelial cells and platelets. Our previous study showed that the binding ability of anti-ΔC NS1 to platelets was lower than that of anti-full-length NS1 Abs [29]. Here, we further showed that the binding activity of anti-ΔC NS1 and anti-DJ NS1 Abs to human endothelial cells (Figure 1B) and to platelets (Figure 1C) were lower than that of anti-DENV NS1 Abs. Therefore, the cross-reactivity of anti-DENV NS1 Abs was reduced after the above-described modifications of the NS1 C-terminus. In this study, we also compared the bleeding times of mice immunized with DENV NS1, ΔC NS1, DJ NS1 or JEV NS1 proteins. The results showed that immunization with unmodified DENV NS1 caused prolonged bleeding time in mice, but immunization with ΔC NS1 and DJ NS1 did not. JEV NS1-immunized mice and normal mice were negative controls (Figure 2). These results suggest that full-length DENV NS1 and its Abs show pathogenic effects but not different versions of C-terminal modified NS1 and their Abs.

### Protective effects of Abs against modified NS1 proteins in a DENV-induced hemorrhagic mouse model

**Figure 3:**
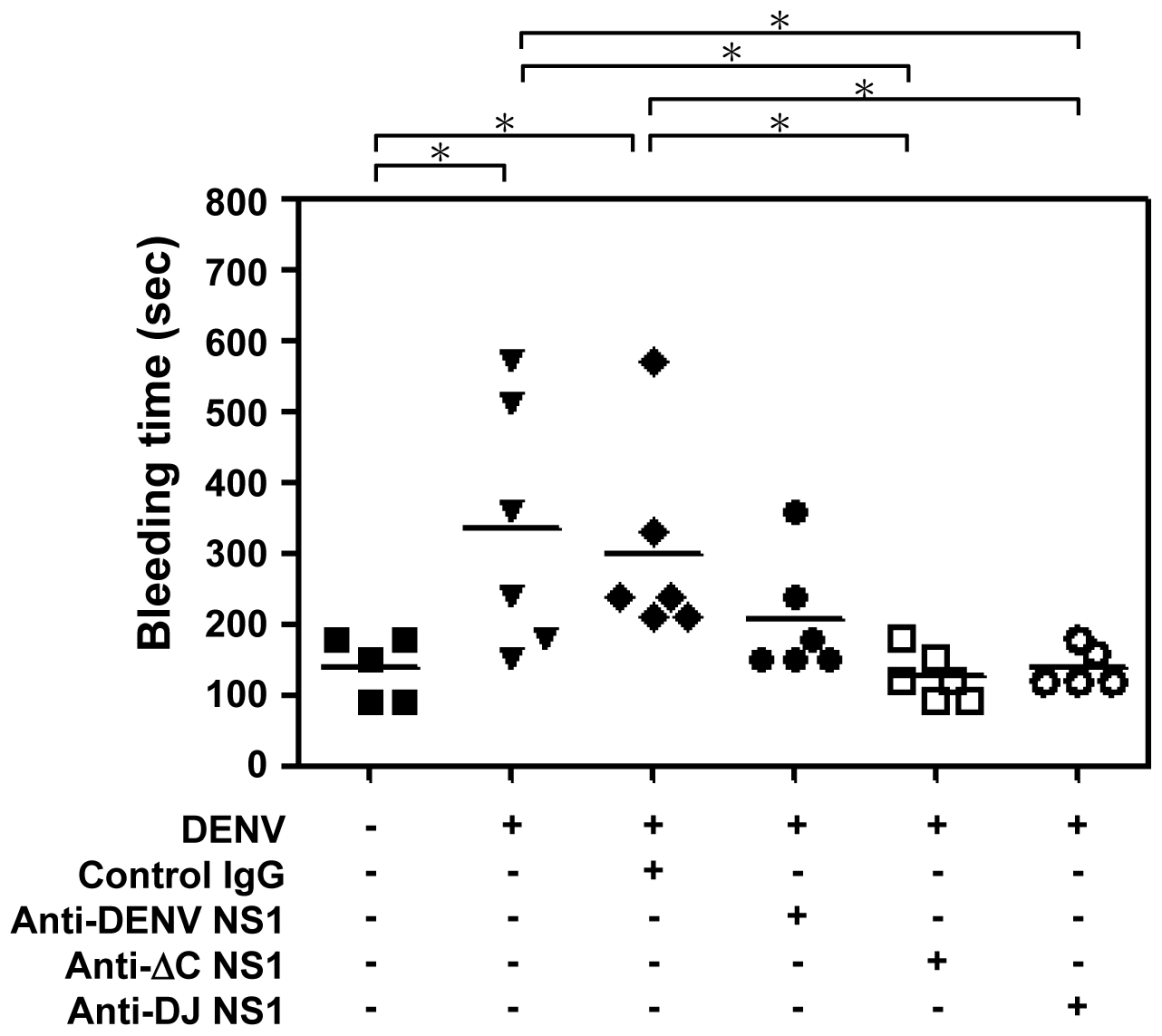
Anti-ΔC NS1 and anti-DJ NS1 Abs reduce DENV-induced prolonged bleeding time in mice. Control IgG, anti-DENV NS1, anti-ΔC NS1 or anti-DJ NS1 Abs (100 μg/mouse) were i.p. injected to C3H/HeN mice, and one day later 9×10^7^ pfu/mouse of DENV were intradermally (i.d.) inoculated in mice. On days one and two post-infection, mice were i.p. injected with 100 μg Abs and the bleeding time was determined on day 3. (n = 5 for medium control and anti-DJ NS1 Abs groups, n = 6 for DENV infection alone, control IgG, anti-DENV NS1 and anti-ΔC NS1 Abs groups) * *P*<0.05.

A mouse model of DENV-induced hemorrhage using intradermal inoculation of virus was previously established [6]-[8]. The hemorrhagic mice showed manifestations of severe thrombocytopenia, prolonged bleeding time, and increased numbers of circulating endothelial cells and infiltrating macrophages. To test the protective effects provided by various Abs, DENV-challenged mice were passively administered with anti-DENV NS1, anti-ΔC NS1 or anti-DJ NS1 Abs. Results showed that while mice infected with DENV alone or passively immunized with normal control IgG showed prolonged bleeding time, passive immunization with anti-ΔC NS1 or anti-DJ NS1 Abs reduced the bleeding time (Figure 3). Passive immunization with anti-full-length DENV NS1 Abs did not cause a significant reduction of bleeding time as compared with the control group. A recent report demonstrated that Abs from dengue patient sera bind to thrombin and inhibit its activity, thereby possibly contributing to the development of hemorrhage in patients with DHF [34]. Therefore, we determined the thrombin activity in our DENV-induced hemorrhagic mouse model. Results showed that the thrombin activity in mice treated with DENV plus control IgG was significantly reduced comparing with the uninfected mice. DENV-infected mice treated with anti-ΔC NS1 and anti-DJ NS1 Abs, but not with anti-DENV NS1 Abs, could partially rescue the inhibition of thrombin activity (Figure S1). These data indicate that anti-ΔC NS1 or anti-DJ NS1 Abs reduce DENV-induced prolonged bleeding time and reverse DENV-reduced thrombin activity. However, anti-full-length DENV NS1 Abs did not reduce or reverse these effects.

**Figure 4:**
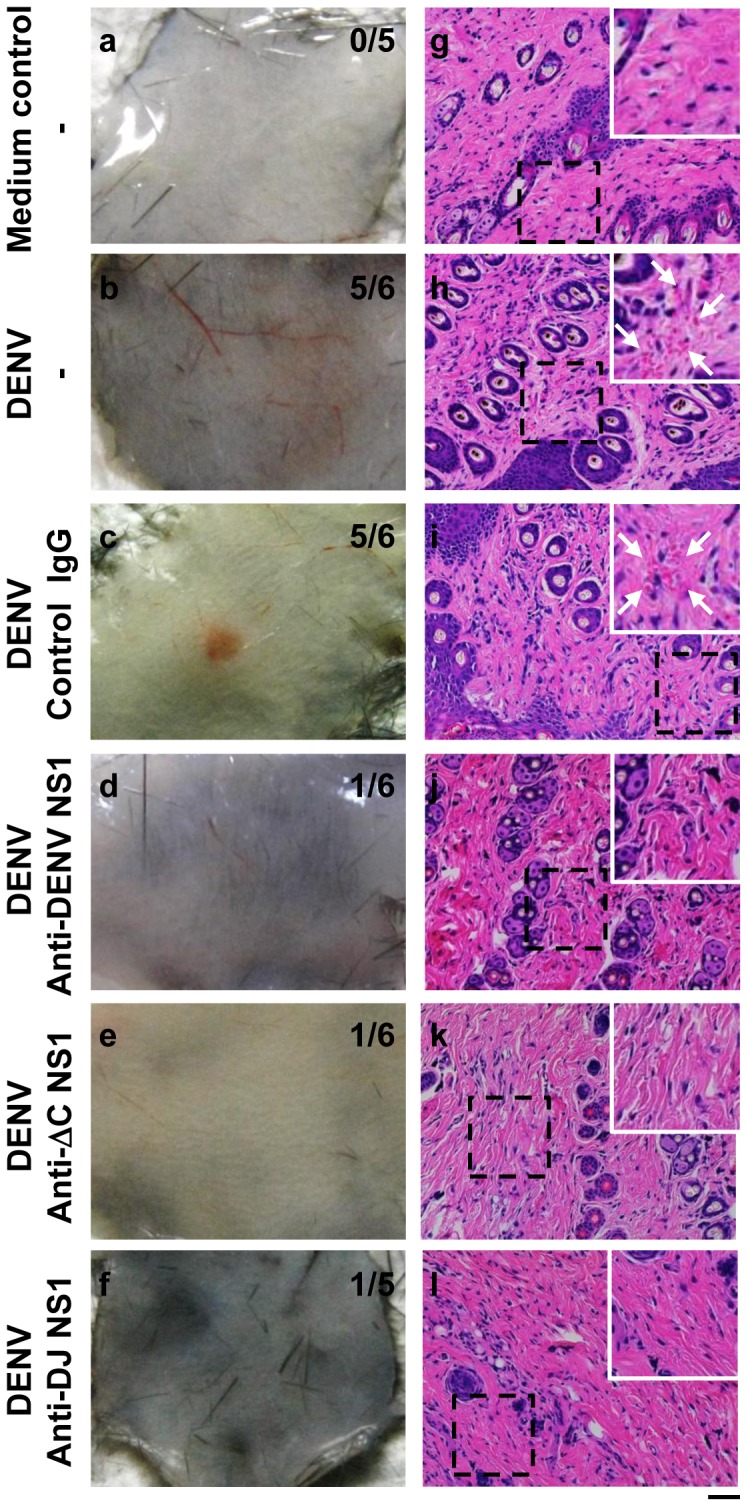
Anti-DENV NS1, anti-ΔC NS1 and anti-DJ NS1 Abs reduce DENV-induced hemorrhage in mice. (a)–(f) show skin samples from mice. The numbers of mice with hemorrhage/total numbers of mice inoculated in each group are indicated. (g)–(l) show the red blood cell extravasation in skin sections. The arrows indicate the regions of red blood cell extravasation. (Magnification: ×200; bar = 100 μm)

Next, we observed hemorrhage development in the mouse skin tissues. Results showed that anti-DENV NS1 (Figure 4d), anti-ΔC NS1 (Figure 4e) and anti-DJ NS1 (Figure 4f) Abs reduced DENV-induced hemorrhage in the skin as compared with mice inoculated with DENV alone (Figure 4b) or mice treated with control IgG (Figure 4c). Mice inoculated with culture medium were used as negative control (Figure 4a). Histopathological examination of the skin sections showed that mice inoculated with DENV alone (Figure 4h) or with control IgG (Figure 4i) displayed red blood cell extravasation, which was not observed in mice treated with anti-DENV NS1 (Figure 4j), anti-ΔC NS1 (Figure 4k) or anti-DJ NS1 (Figure 4l). Mice inoculated with culture medium were used as the negative control to show that simple intradermal injections did not lead to hemorrhage development (Figure 4a, g). These data demonstrate that anti-DENV NS1, anti-ΔC NS1 or anti-DJ NS1 Abs reduce DENV-induced hemorrhage.

**Figure 5:**
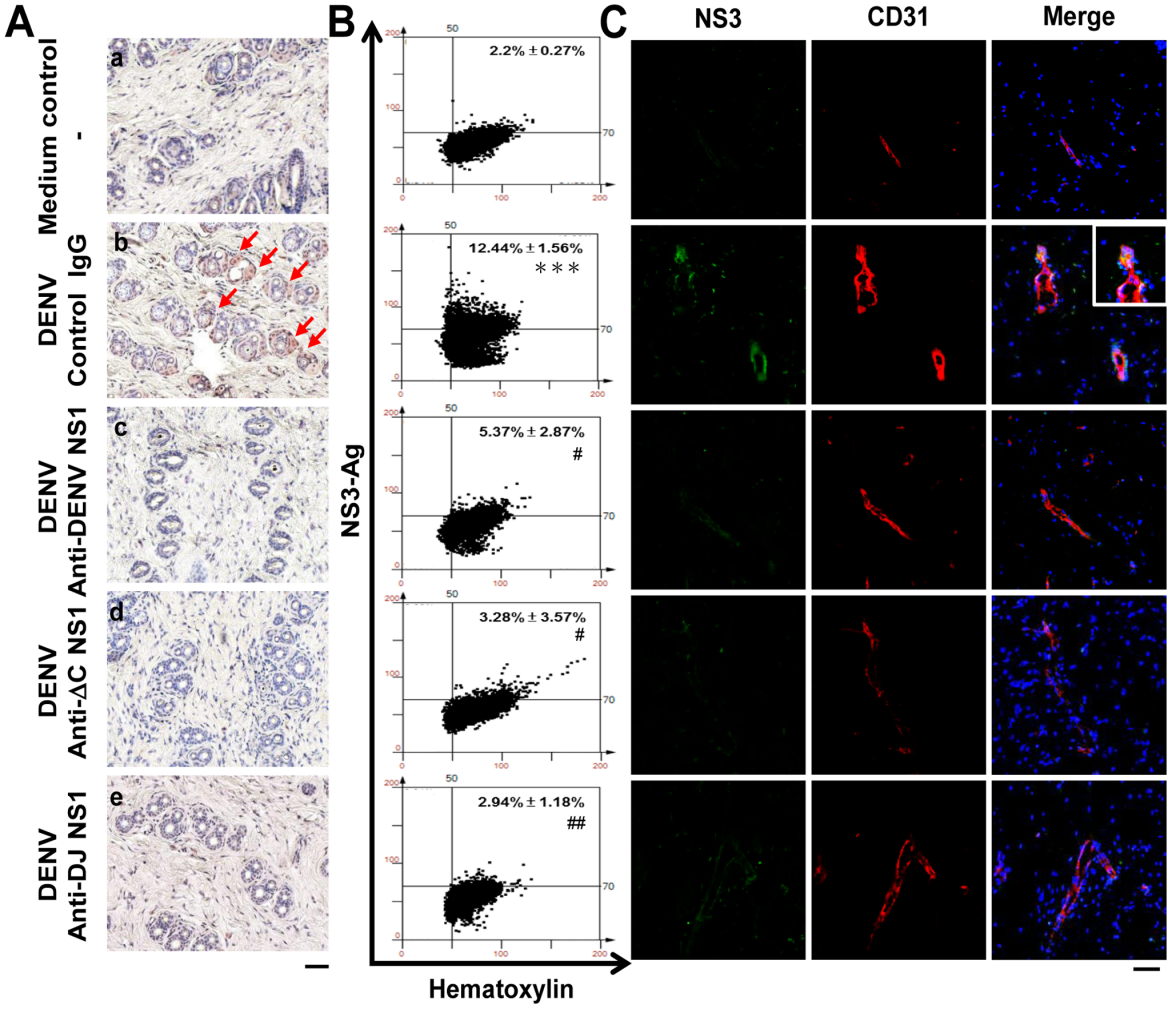
Anti-DENV NS1, anti-ΔC NS1 and anti-DJ NS1 Abs reduce DENV NS3 expression. (A) The local skin was collected and fixed in paraffin. The skin sections were stained with anti-DENV NS3 Abs, followed by HRP-conjugated anti-rabbit IgG. Nuclei were stained with hematoxylin (blue). The arrows indicate positive staining. (a)–(e) show the NS3 expression of cells in the dermis layer of skin sections. (Magnification: ×200; bar = 100 μm) (B) Quantification of NS3 staining was performed on skin sections using HistoQuest analysis software. A representative scattergram plot of each group is shown and the percentage of NS3-positive cells is shown as means ± SD obtained from three mice of each group. *** *P*<0.001 as compared with medium control group. ^#^ *P*<0.05; ^##^ *P*<0.01 as compared with DENV plus control IgG group. (C) Skin cryosections were stained with anti-DENV NS3 and CD31 Abs, followed by Alexa-488-conjugated anti-rabbit IgG and Alexa-594-conjugated anti-rat IgG. Nuclei were stained with DAPI. (Magnification: ×200; bar = 50 μm)

We next checked the presence of DENV NS3, a marker for viral replication, in the mouse skin sections. Immunohistochemical staining showed that cells in the dermis layer (Figure 5A, b) expressed NS3 in mice treated with DENV plus control IgG. However, anti-DENV NS1 (Figure 5A, c), anti-ΔC NS1 (Figure 5A, d) and anti-DJ NS1 (Figure 5A, e) Abs reduced DENV antigens in the skin tissues. Mice inoculated with culture medium were used as the negative control (Figure 5A, a). The images of immunohistochemical staining were further quantified. Results of HistoQuest analysis show that DENV-infected mice treated with anti-ΔC NS1 and anti-DJ NS1 Abs as well as anti-DENV NS1 Abs significantly reduced the levels of viral NS3 in infected mice (Figure 5B). Immunofluorescence staining also showed that CD31^+^ vascular endothelial cells in the skin sections expressed NS3 in mice treated with DENV plus control IgG. In contrast, DENV-infected mice treated with anti-ΔC NS1 and anti-DJ NS1 Abs as well as anti-DENV NS1 Abs reduced the levels of viral NS3 in CD31^+^ endothelial cells (Figure 5C).

### Abs against modified NS1 proteins induce complement-mediated cytotoxicity in DENV-infected endothelial cells and inhibit viral replication

**Figure 6:**
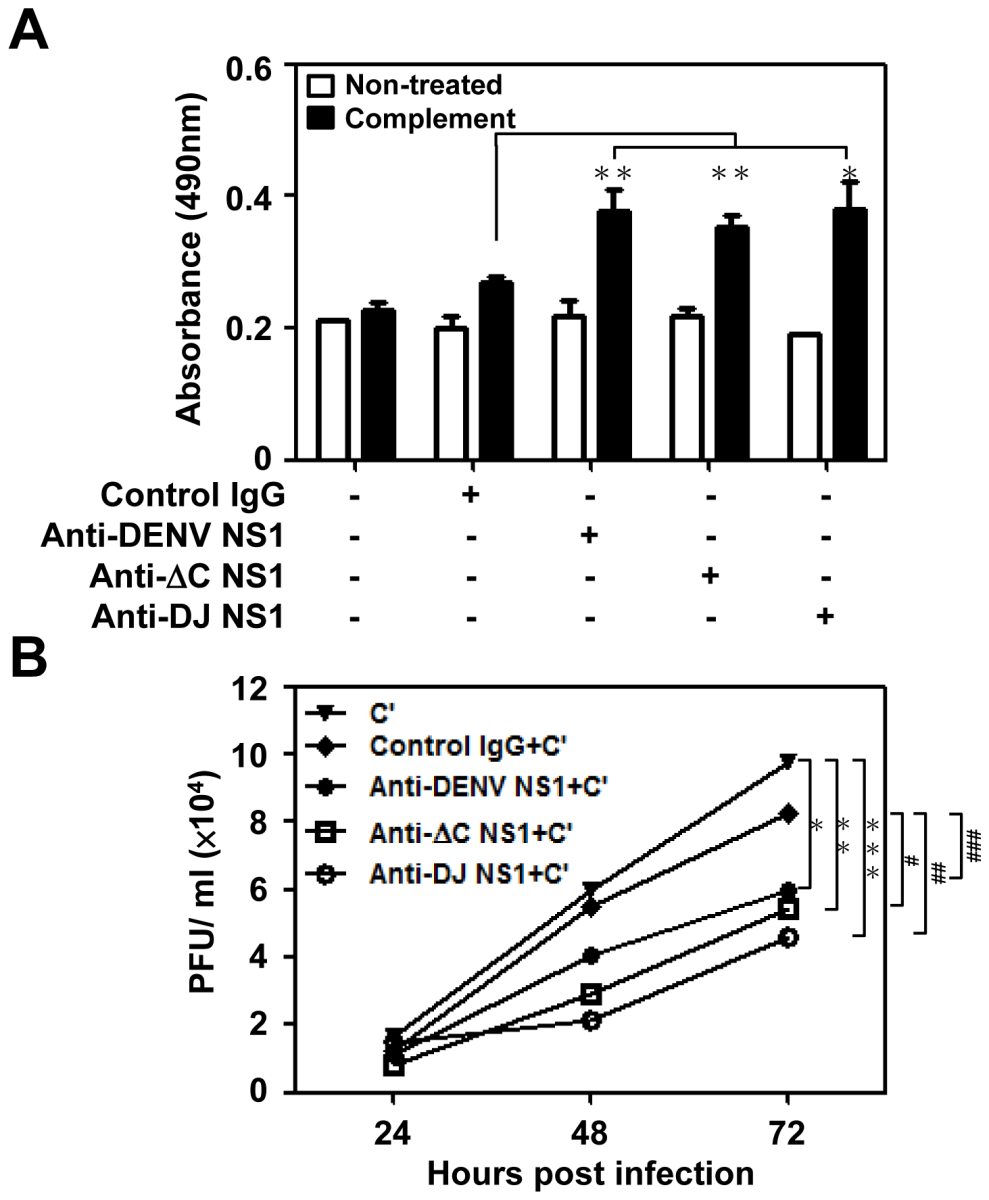
Anti-DENV NS1, anti-ΔC NS1 and anti-DJ NS1 Abs cause complement-mediated cytolysis and inhibit virus release into the culture medium. (A) HMEC-1 cells were infected with DENV (MOI = 10) for 72 h. At 72 h post-infection, cells were incubated with control IgG, anti-DENV NS1, anti-ΔC NS1 or anti-DJ NS1 Abs (50 μg/ml) for 1 h at 4°C and incubated with or without complement for 4 h at 37°C. Cell culture supernatants were collected and detected for the release of lactate dehydrogenase (LDH). * *P*<0.05; ** *P*<0.01. (B) At 6 h post-infection, DENV-infected HMEC-1 cells were incubated with control IgG, anti-DENV NS1, anti-ΔC NS1 or anti-DJ NS1 Abs (50 μg/ml) in the presence of complement (C′) and cultured at 37°C. Virus titers in the culture medium at the indicated time points were measured by plaque assay. The averages of triplicate cultures are shown. * *P*<0.05; ** *P*<0.01; *** *P*<0.001 as compared with C′ alone group. ^#^ *P*<0.05; ^##^ *P*<0.01; ^###^ *P* = 0.076 as compared with C′ plus control IgG group.

Several studies suggested that flavivirus NS1-specific Abs kill infected cells in a complement-dependent manner [35]-[38]. In DENV infection, complement activation is triggered by Abs binding to the NS1 proteins expressed on the surface of infected cells [16], [18]. We confirmed the enhanced binding ability of anti-DENV NS1, ΔC NS1 and DJ NS1 Abs to DENV-infected endothelial cells. These results indicate that these Abs can bind the NS1 protein expressed on the cell surface by DENV infection (Figure S2). To determine whether these Abs can cause cytolysis of DENV-infected cells in the presence of complement, a complement-mediated cytotoxicity assay was performed. Compared to control IgG treatment, treatment with anti-ΔC NS1, anti-DJ NS1 and anti-DENV NS1 Abs, had the ability to lyse DENV-infected cells in the presence of complement as measured by the release of LDH (Figure 6A). A previous study demonstrated that anti-JEV NS1 Abs caused a complement-dependent reduction in virus output from infected human cells, indicating an important role in viral control [37]. To test the effect of Abs and complement on DENV replication, DENV-infected endothelial cells were treated with anti-DENV NS1, anti-ΔC NS1 or anti-DJ NS1 Abs plus complement. Virus in the culture medium at the indicated time points was measured by plaque assay. At 72 h post-infection, there was a significant reduction in virus titer observed in the presence of anti-ΔC NS1 and anti-DJ NS1 Abs, as compared to control IgG plus complement or complement alone (Figure 6B). These results indicate that anti-ΔC NS1 and anti-DJ NS1 Abs induce complement-mediated cytolysis of cells which express NS1 proteins on their surface and are consequently unable to support viral replication.

### Abs against modified NS1 proteins reduce macrophage infiltration in DENV-induced hemorrhagic mouse model

**Figure 7:**
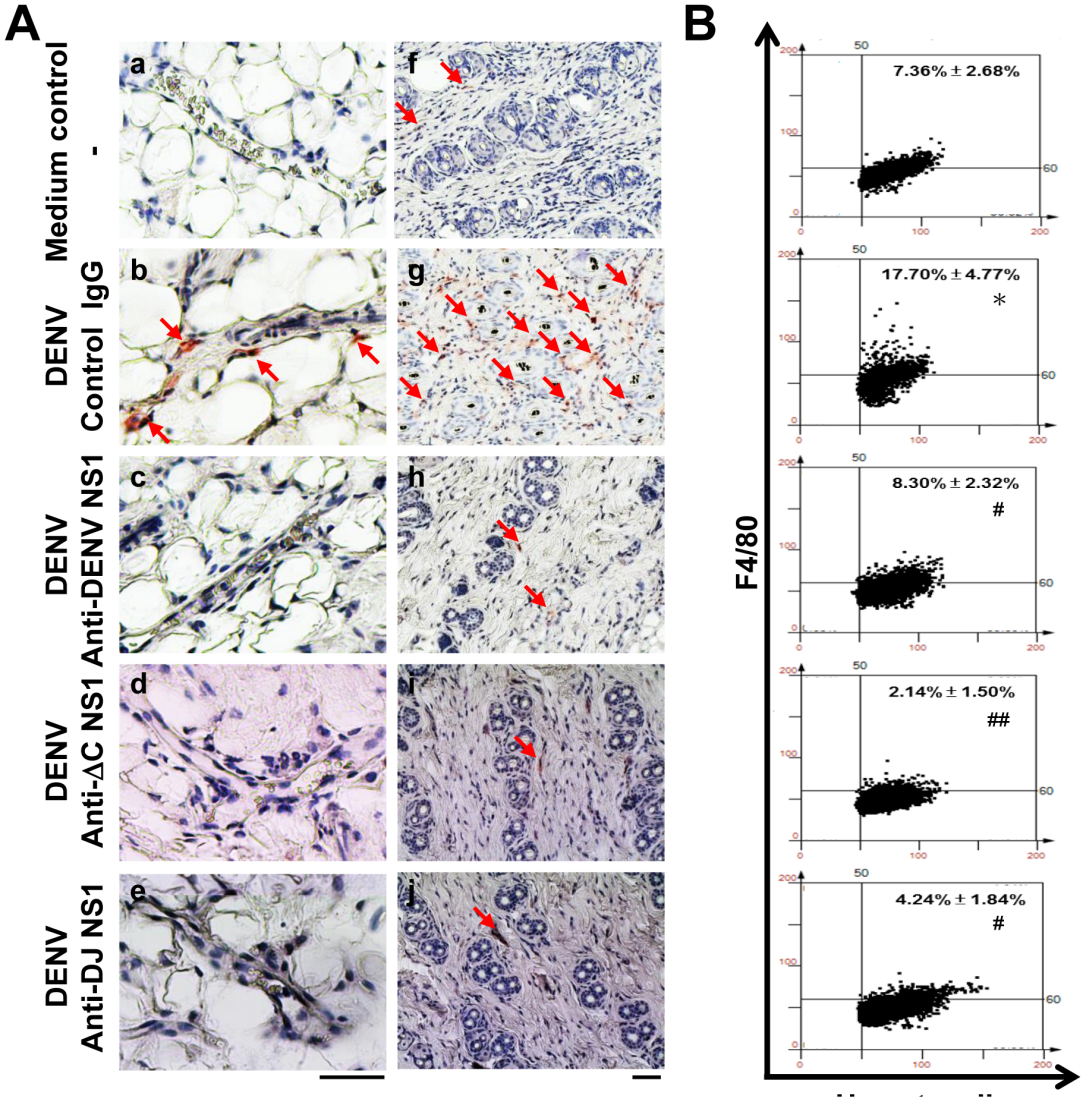
Anti-DENV NS1, anti-ΔC NS1 and anti-DJ NS1 Abs reduce macrophage infiltration in DENV-infected mice. (A) Hematoxylin-based nuclear staining followed by immunohistochemical staining of F4/80, a macrophage marker, in skin vessel (a–e) and dermis layer (f–j) of treated mice were observed. The arrows indicate positive staining. (Magnification: ×200; bar = 100 μm) (B) Quantification of F4/80 staining was performed on skin sections using HistoQuest analysis software. A representative scattergram plot of each group is shown and the percentage of F4/80 positive cells is shown as means ± SD obtained from three mice of each group. * *P*<0.05 as compared with medium control group. ^#^ *P*<0.05; ^##^ *P*<0.01 as compared with DENV plus control IgG group.

Endothelial cell damage or activation is accompanied by infiltrating macrophages and circulating monocytes that secrete TNF-α [7], [39]. In this study, immunohistochemistry staining showed that mice inoculated with DENV plus control IgG revealed close contact of F4/80-positive macrophages to endothelium (Figure 7A, b). In addition, F4/80-positive macrophages were also observed infiltrating to the dermis layer (Figure 7A, g). In contrast, there were no or only few infiltrating macrophages in the endothelium layer (Figure 7A, c-e) and the dermis (Figure 7A, h-j) in mice treated with anti-DENV NS1, anti-ΔC NS1 or anti-DJ NS1 Abs. Mice inoculated with culture medium were used as the negative control showing no or only few infiltrating macrophages close to the endothelium and in the dermis layer (Figure 7A, a, f). The images of immunohistochemical staining were further quantified using HistoQuest analysis software. Treatment with anti-ΔC NS1 and anti-DJ NS1 Abs as well as anti-DENV NS1 Abs showed significant reduction of infiltrating macrophages (Figure 7B). These results suggest that anti-DENV NS1, anti-ΔC NS1 or anti-DJ NS1 Abs reduce DENV-induced inflammation by inhibiting macrophage infiltration.

### Active immunization with modified NS1 provides protection in the DENV-induced hemorrhagic mouse model

**Figure 8:**
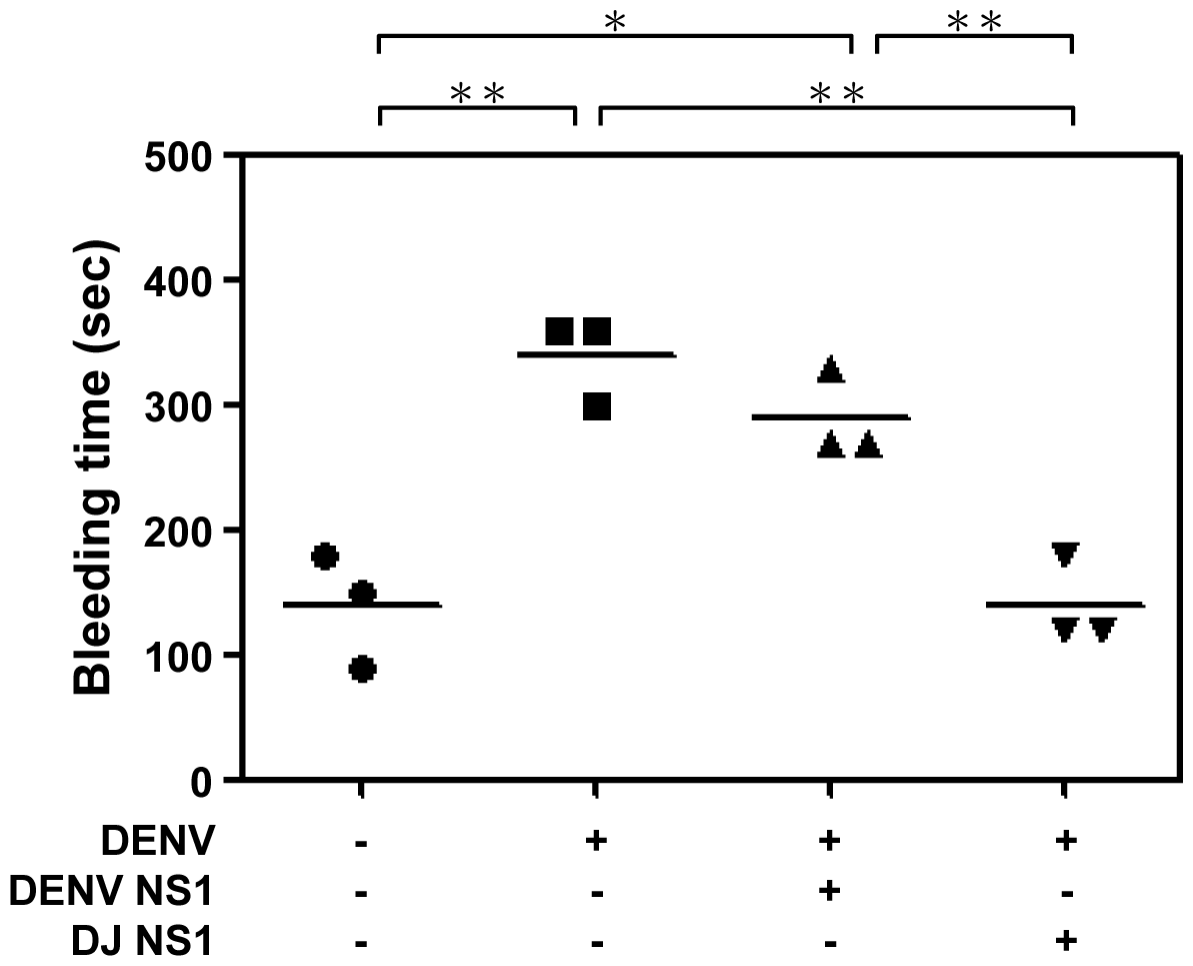
Active immunization with DJ NS1 decreases the DENV-induced prolonged bleeding time. C3H/HeN mice (n = 3/group) were i.p. immunized three times with DENV NS1 or DJ NS1 proteins and three days later mice were i.d. inoculated with 9×10^7^ pfu/mouse of DENV. The bleeding time was determined on day 3 post-infection. * *P*<0.05, ** *P*<0.01.

To further test the protective efficacy of modified NS1 proteins, we used active immunization protocols in the mouse model. Mice were actively immunized with DENV NS1 or DJ NS1 proteins before DENV challenge. The results showed that active immunization with DJ NS1 significantly reduced the DENV-induced prolonged bleeding time to a level similar to the uninfected group. However, active immunization with DENV NS1 did not reduce the DENV-induced prolonged bleeding time (Figure 8).

**Figure 9:**
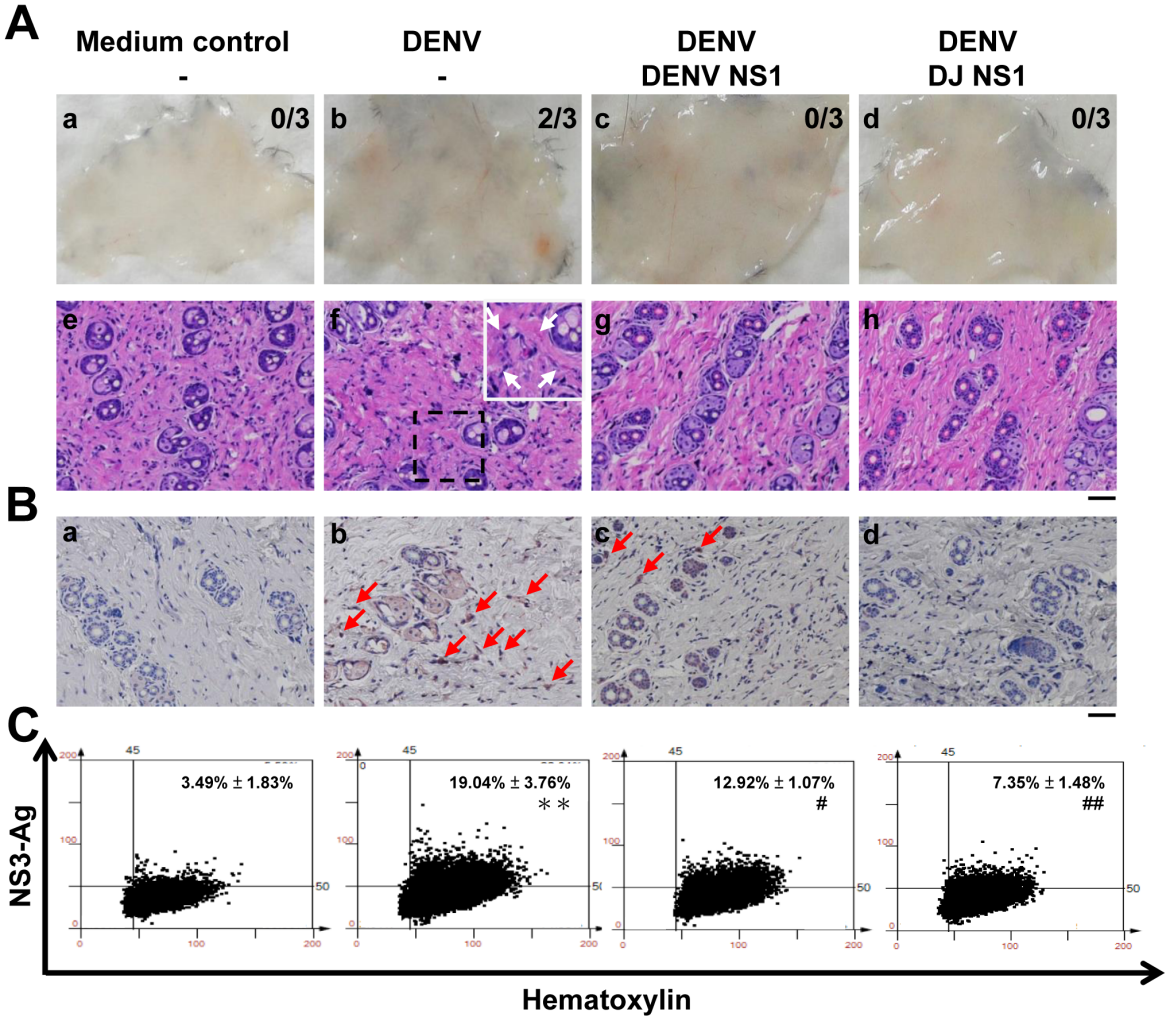
Active immunization with DJ NS1 reduces the DENV-induced hemorrhage and DENV NS3 expression in mice. (A) (a)–(d) show skin samples from mice. The numbers of mice with hemorrhage/total numbers of mice inoculated in each group are indicated. (e)–(h) show the red blood cell extravasation in skin sections. The arrows indicate the regions of red blood cell extravasation. (Magnification: ×200; bar = 100 μm) (B) The local skin was collected and fixed in paraffin. The skin sections were stained with anti-DENV NS3 Abs, followed by HRP-conjugated anti-rabbit IgG. Nuclei were stained with hematoxylin (blue). The arrows indicate positive staining. (a)–(d) show the NS3 expression of cells in the dermis layer of skin sections. (Magnification: ×200; bar = 100 μm) (C) Quantification of NS3 staining was performed on skin sections using HistoQuest analysis software. A representative scattergram plot of each group is shown and the percentage of NS3 positive cells is shown as means ± SD obtained from three mice of each group. ** *P*<0.01 as compared with medium control group. ^#^ *P* = 0.053; ^##^ *P*<0.01 as compared with DENV alone group.

Next, we observed both hemorrhage development and DENV NS3 levels in the mouse skin tissues. Results showed that DENV NS1 (Figure 9A, c) and DJ NS1 (Figure 9A, d) immunization reduced DENV-induced hemorrhage in the skin as compared with mice inoculated with DENV alone (Figure 9A, b). Mice inoculated with culture medium were used as negative control (Figure 9A, a). Histopathological examination of the skin sections showed that mice inoculated with DENV alone (Figure 9A, f) displayed red blood cell extravasation, which was not observed in mice immunized with DENV NS1 (Figure 9A, g) or DJ NS1 (Figure 9A, h) before DENV challenge. Mice inoculated with culture medium were used as a negative control to show that intradermal injection itself did not lead to hemorrhage development (Figure 9A, e). Immunohistochemical staining showed that cells in the dermis layer expressed NS3 in mice inoculated with DENV (Figure 9B, b). Active immunization with DJ NS1 protein significantly reduced DENV NS3 antigen expression in the skin tissues (Figure 9B, d), whereas active immunization with DENV NS1 protein showed less reduction of the levels of viral NS3 in infected mice (Figure 9B, c). Mice inoculated with culture medium were used as a negative control (Figure 9B, a). The images of immunohistochemical staining were further quantified by HistoQuest analysis (Figure 9C). Taken together, these results indicate that DJ NS1 exhibits lower pathogenic effects and is a better candidate than unmodified DENV NS1 for future dengue vaccine development.

## Discussion

This is the first study demonstrating vaccine potential for Abs against a modified DENV NS1 protein, from which harmful cross-reactive epitopes have been selectively deleted. Although the NS1 protein is not a virion structural protein, Abs against it can protect against infection of several flaviviruses *in vivo*, such as Yellow Fever Virus [35], [40], JEV [36], [41], West Nile Virus [42], [43] and DENV [16], [18]-[20], [44]. Abs against NS1 exhibit cytolytic activity to kill virus-infected cells in a complement-dependent manner. In the present study, we demonstrate that not only anti-DENV NS1 Abs but also anti-ΔC NS1 and anti-DJ NS1 Abs possess the ability of Ab-mediated complement-dependent cytotoxicity. Moreover, all three Abs have the ability to control viral load *in vivo* (Figure 5) and *in vitro* (Figure 6B). A previous report showed that anti-NS1 Abs trigger Fcγ receptor-mediated phagocytosis and clearance of West Nile Virus-infected cells [45]. Therefore, in addition to Ab-mediated complement-dependent cytotoxicity, other mechanisms by which anti-ΔC NS1 and anti-DJ NS1 Abs contribute to protection from DENV challenge need to be further investigated.

Anti-full-length NS1 exhibited higher endothelial cell or platelet binding activity than that of anti-ΔC NS1 and anti-DJ NS1 Abs (Figure 1B and C). In addition, anti-DENV NS1 Abs, in the presence of complement, cause cytolysis of uninfected endothelial cells due to cross-reactive effects (Figure S3). These results suggest that full-length DENV NS1 and its Abs show undesirable pathogenic effects but not the various forms of C-terminal modified NS1 and their Abs. Although all three Abs show similar protective effects at local infection sites or in DENV-infected cell culture (Figure 4, 5, 6, 7), the DENV-induced prolonged bleeding time was markedly reduced when mice were treated with anti-ΔC NS1 and anti-DJ NS1 Abs whereas the reduction in bleeding time mediated by anti-full-length DENV NS1 Abs was not statistically significant (Figure 3). Furthermore, active immunization with DJ NS1, but not DENV NS1, significantly reduced the DENV-induced prolonged bleeding time (Figure 8), hemorrhage development, and DENV NS3 antigen levels (Figure 9). Therefore, the various forms of C-terminal modified NS1 and their Abs provide better protection against virus challenge than full-length DENV NS1 and its Abs by reducing the risk of pathogenic effects. Although anti-ΔC NS1 and anti-DJ NS1 Abs show similar protective effects, further studies are necessary to discriminate possible differences in protein conformation and antigenicity.

Previous studies in our laboratory identified major cross-reactive epitopes in the C-terminus (a.a. 271-352) of DENV NS1 [28], [29], [33], [46]. Recent studies also indicated that a.a. 116-119 of DENV NS1 share similar sequences with human LYRIC (a.a. 334-337) [23]. Also, Abs generated to ELK/KLE-type motifs in the NS1 reacted with blood blotting and adhesion molecules [21], [22]. Therefore, these and possibly other epitopes should be further characterized to decrease the risk of cross-reactivity from a vaccine safety perspective.

So far no licensed dengue vaccines are available, but there are some successful vaccines for other flaviviruses such as Yellow Fever Virus or JEV [47]. One of the reasons is that, unlike other monotypic flaviviruses, there are four serotypes of DENV. Even worse, anti-DENV Abs, especially anti-E or anti-prM Abs, not only do not provide cross-protection against other serotypes of DENV but also enhance the infection [12]–[14]. Therefore, many efforts have been directed at developing tetravalent vaccines against dengue [48]–[52]. One of the leading candidates, the Sanofi dengue vaccine, showed unequal efficacy against the four serotypes of DENV [53]. Much investigation is required to determine whether NS1 or modified NS1 proteins can provide protection against different serotypes.

An important aspect of our study is the use of dengue hemorrhage mouse model to evaluate the potential vaccine candidates. It was previously shown that NS1 immunization can prevent mortality caused by intracerebral inoculation of DENV [16], [18], [44]. The dengue hemorrhage mouse model we used in the present study mimicked the natural route of infection in humans. Infected mice developed hemorrhage and had prolonged bleeding time. Here we showed that passive immunization with Abs against ΔC NS1 and DJ NS1 were protective against DENV-induced hemorrhage. While recent reports demonstrated that Abs against DENV E protein [54], [55] and NS1 [56] represent new therapeutic candidates for treating DENV infection, our results show further that Abs against modified NS1 protein may provide additional therapeutic benefits by reducing potentially harmful cross-reactive effects.

In conclusion, we have shown in the present study the protective effects provided by Abs against ΔC NS1 and DJ NS1 proteins using a dengue hemorrhagic mouse model. Unlike anti-full-length DENV NS1 Abs, anti-ΔC NS1 and anti-DJ NS1 Abs do not cross-react with uninfected endothelial cells and platelets, and do not cause prolonged bleeding time in mice. Our findings suggest that C-terminal modification of NS1 proteins may be an attractive strategy for dengue vaccine development.

## Supporting Information

Figure S1 **Anti-ΔC NS1 and anti-DJ NS1 Abs rescue DENV-induced thrombin activity inhibition.** Thrombin activity was detected by adding chromogenic substrates S-2238 to the mouse plasma and incubating at room temperature for 1 h. The optical density value at 405 nm was measured every 10 min with a VersaMax microplate reader [34]. The inhibition of thrombin activity occurred in the DENV plus control IgG group compared to medium control and was partially rescued by anti-ΔC NS1 and anti-DJ NS1 Abs treatment (n = 4/group). * *P*<0.05.
(TIF)

Figure S2 **Anti-ΔC NS1 and anti-DJ NS1 Abs possess binding activity to DENV-infected endothelial cells.** HMEC-1 cells were infected with DENV (MOI = 10) for 48 or 72 h. Non-fixed cells were stained with control IgG, anti-DENV NS1, anti-ΔC NS1 or anti-DJ NS1 Abs (2.5 μg), followed by Alexa 488-conjugated donkey anti-mouse IgG staining and analyzed by flow cytometry. The averages ± SD obtained from triplicate cultures are shown.
(TIF)

Figure S3 **Anti-DENV NS1 Abs cause complement-mediated cytolysis in uninfected cells.** Uninfected HMEC-1 cells were incubated with control IgG, anti-DENV NS1, anti-ΔC NS1 or anti-DJ NS1 Abs (50 μg/ml) for 1 h at 4°C and then incubated with or without complement for 4 h at 37°C. Cell culture supernatants were collected and detected for the release of lactate dehydrogenase (LDH). The averages ± SD obtained from triplicate cultures are shown. * *P*<0.05.
(TIF)

## References

[1] Guzman MG, Halstead SB, Artsob H, Buchy P, Farrar J, et al (2010) Dengue: a continuing global threat. Nat Rev Microbiol 8: S7–S16. 21079655 10.1038/nrmicro2460 PMC4333201

[2] Bhatt S, Gething PW, Brady OJ, Messina JP, Farlow AW, et al (2013) The global distrubution and burden of dengue. Nature 496: 504–507. 23563266 10.1038/nature12060 PMC3651993

[3] Simmons CP, Farrar JJ, Nguyen vV, Wills B (2012) Dengue. N Engl J Med 366: 1423–1432. 22494122 10.1056/NEJMra1110265

[4] Halstead SB (2007) Dengue. Lancet 370: 1644–1652. 17993365 10.1016/S0140-6736(07)61687-0

[5] Yauch LE, Shresta S (2008) Mouse models of dengue virus infection and disease. Antiviral Res 80: 87–93. 18619493 10.1016/j.antiviral.2008.06.010 PMC3048811

[6] Chen HC, Hofman FM, Kung JT, Lin YD, Wu-Hsieh BA (2007) Both virus and tumor necrosis factor alpha are critical for endothelium damage in a mouse model of dengue virus-induced hemorrhage. J Virol 81: 5518–5526. 17360740 10.1128/JVI.02575-06 PMC1900309

[7] Yen YT, Chen HC, Lin YD, Shieh CC, Wu-Hsieh BA (2008) Enhancement by tumor necrosis factor alpha of dengue virus-induced endothelial cell production of reactive nitrogen and oxygen species is key to hemorrhage development. J Virol 82: 12312–12324. 18842737 10.1128/JVI.00968-08 PMC2593312

[8] Wu-Hsieh BA, Yen YT, Chen HC (2009) Dengue hemorrhage in a mouse model. Ann N Y Acad Sci 1171 Suppl 1E42–E47. 19751401 10.1111/j.1749-6632.2009.05053.x

[9] Halstead SB (1998) Pathogenesis of dengue: challenges to molecular biology. Science 239: 476–481. 10.1126/science.3277268 3277268

[10] Morens DM (1994) Antibody-dependent enhancement of infection and the pathogenesis of viral disease. Clin Infect Dis 19: 500–512. 7811870 10.1093/clinids/19.3.500

[11] Halstead SB (2002) Dengue. Curr Opin Infect Dis 15: 471–476. 12686878 10.1097/00001432-200210000-00003

[12] Halstead SB (2003) Neutralization and antibody-dependent enhancement of dengue viruses. Adv Virus Res 60: 421–467. 14689700 10.1016/s0065-3527(03)60011-4

[13] Huang KJ, Yang YC, Lin YS, Huang JH, Liu HS, et al (2006) The dual-specific binding of dengue virus and target cells for the antibody-dependent enhancement of dengue virus infection. J Immunol 176: 2825–2832. 16493039 10.4049/jimmunol.176.5.2825

[14] Goncalvez AP, Engle RE, St Claire M, Purcell RH, Lai CJ (2007) Monoclonal antibody-mediated enhancement of dengue virus infection in vitro and in vivo and strategies for prevention. Proc Natl Acad Sci USA 104: 9422–9427. 17517625 10.1073/pnas.0703498104 PMC1868655

[15] Balsitis SJ, Williams KL, Lachica R, Flores D, Kyle JL, et al (2010) Lethal antibody enhancement of dengue disease in mice is prevented by Fc modification. PLoS Pathog 6: e1000790. 20168989 10.1371/journal.ppat.1000790 PMC2820409

[16] Schlesinger JJ, Brandriss MW, Walsh EE (1987) Protection of mice against dengue 2 virus encephalitis by immunization with the dengue 2 virus non-structural glycoprotein NS1. J Gen Virol 68 (Pt 3): 853–857. 10.1099/0022-1317-68-3-853 3819700

[17] Amorim JH, Diniz MO, Cariri FA, Rodrigues JF, Bizerra RS, et al (2012) Protective immunity to DENV2 after immunization with a recombinant NS1 protein using a genetically detoxified heat-labile toxin as an adjuvant. Vaccine 30: 837–845. 22178517 10.1016/j.vaccine.2011.12.034

[18] Henchal EA, Henchal LS, Schlesinger JJ (1998) Synergistic interactions of anti-NS1 monoclonal antibodies protect passively immunized mice from lethal challenge with dengue 2 virus. J Gen Virol 69 (Pt 8): 2101–2107. 10.1099/0022-1317-69-8-2101 3404125

[19] Wu SF, Liao C L, Lin YL, Yeh CT, Chen LK, et al (2003) Evaluation of protective efficacy and immune mechanisms of using a non-structural protein NS1 in DNA vaccine against dengue 2 virus in mice. Vaccine 21: 3919–3929. 12922127 10.1016/s0264-410x(03)00310-4

[20] Costa SM, Azevedo AS, Paes MV, Sarges FS, Freire MS, et al (2007) DNA vaccines against dengue virus based on the ns1 gene: the influence of different signal sequences on the protein expression and its correlation to the immune response elicited in mice. Virology 358: 413–423. 17020777 10.1016/j.virol.2006.08.052

[21] Falconar AK (1997) The dengue virus nonstructural-1 protein (NS1) generates antibodies to common epitopes on human blood clotting, integrin/adhesin proteins and binds to human endothelial cells: potential implications in haemorrhagic fever pathogenesis. Arch Virol 142: 897–916. 9191856 10.1007/s007050050127

[22] Falconar AK (2007) Antibody responses are generated to immunodominant ELK/KLE-type motifs on the nonstructural-1 glycoprotein during live dengue virus infections in mice and humans: implications for diagnosis, pathogenesis, and vaccine design. Clin Vaccine Immunol 14: 493–504. 17329445 10.1128/CVI.00371-06 PMC1865631

[23] Liu IJ, Chiu CY, Chen YC, Wu HC (2011) Molecular mimicry of human endothelial cell antigen by autoantibodies to nonstructural protein 1 of dengue virus. J Biol Chem 286: 9726–9736. 21233208 10.1074/jbc.M110.170993 PMC3058979

[24] Lin YS, Yeh TM, Lin CF, Wan SW, Chuang YC, et al (2011) Molecular mimicry between virus and host and its implications for dengue disease pathogenesis. Exp Biol Med 236: 515–523. 10.1258/ebm.2011.010339 21502191

[25] Lin CF, Lei HY, Liu CC, Liu HS, Yeh TM, et al (2008) Patient and mouse antibodies against Dengue virus nonstructural protein 1 cross-react with platelets and cause their dysfunction or depletion. Am J Infect Dis 4: 69–75.

[26] Lin CF, Lei HY, Shiau AL, Liu HS, Yeh TM, et al (2002) Endothelial cell apoptosis induced by antibodies against dengue virus nonstructural protein 1 via production of nitric oxide. J Immunol 169: 657–664. 12097367 10.4049/jimmunol.169.2.657

[27] Lin CF, Chiu SC, Hsiao YL, Wan SW, Lei HY, et al (2005) Expression of cytokine, chemokine, and adhesion molecules during endothelial cell activation induced by antibodies against dengue virus nonstructural protein 1. J Immunol 174: 395–403. 15611263 10.4049/jimmunol.174.1.395

[28] Cheng HJ, Lin CF, Lei HY, Liu HS, Yeh TM, et al (2009) Proteomic analysis of endothelial cell autoantigens recognized by anti-dengue virus nonstructural protein 1 antibodies. Exp Biol Med 234: 63–73. 10.3181/0805-RM-147 18997103

[29] Chen MC, Lin CF, Lei HY, Lin SC, Liu HS, et al (2009) Deletion of the C-terminal region of dengue virus nonstructural protein 1 (NS1) abolishes anti-NS1-mediated platelet dysfunction and bleeding tendency. J. Immunol 183: 1797–1803. 19592650 10.4049/jimmunol.0800672

[30] Séverin S, Gratacap MP, Lenain N, Alvarez L, Hollande E, et al (2007) Deficiency of Src homology 2 domain-containing inositol 5-phosphatase 1 affects platelet responses and thrombus growth. J Clin Invest 117: 944–952. 17347685 10.1172/JCI29967 PMC1810573

[31] Ades EW, Candal FJ, Swerlick RA, George VG, Summers S, et al (1992) HMEC-1: establishment of an immortalized human microvascular endothelial cell line. J Invest Dermatol 99: 683–690. 1361507 10.1111/1523-1747.ep12613748

[32] Huang KJ, Li SY, Chen SC, Liu HS, Lin YS, et al (2000) Manifestation of thrombocytopenia in dengue-2-virus-infected mice. J Gen Virol 81: 2177–2182. 10950974 10.1099/0022-1317-81-9-2177

[33] Wan SW, Lin CF, Chen MC, Lei HY, Liu HS, et al (2008) C-terminal region of dengue virus nonstructural protein 1 is involved in endothelial cell cross-reactivity via molecular mimicry. Am J Infect Dis 4: 85–91.

[34] Chuang YC, Lin YS, Liu HS, Wang JR, Yeh TM (2013) Antibodies against thrombin in dengue patients contain both anti-thrombotic and pro-fibrinolytic activities. Thromb Haemost 110: 358–365. 23740201 10.1160/TH13-02-0149

[35] Schlesinger JJ, Brandriss MW, Walsh EE (1985) Protection against 17D yellow fever encephalitis in mice by passive transfer of monoclonal antibodies to the nonstructural glycoprotein gp48 and by active immunization with gp48. J Immunol 135: 2805–2809. 4031501

[36] Lin YL, Chen LK, Liao CL, Yeh CT, Ma SH, et al (1998) DNA immunization with Japanese encephalitis virus nonstructural protein NS1 elicits protective immunity in mice. J Virol 72: 191–200. 9420215 10.1128/jvi.72.1.191-200.1998 PMC109364

[37] Krishna VD, Rangappa M, Satchidanandam V (2009) Virus-specific cytolytic antibodies to nonstructural protein 1 of Japanese encephalitis virus effect reduction of virus output from infected cells. J Virol 83: 4766–4777. 19264772 10.1128/JVI.01850-08 PMC2682061

[38] Kitai Y, Kondo T, Konishi E (2010) Complement-dependent cytotoxicity assay for differentiating West Nile virus from Japanese encephalitis virus infections in horses. Clin Vaccine Immunol 17: 875–878. 20237201 10.1128/CVI.00217-09 PMC2863385

[39] Anderson R, Wang S, Osiowy C, Issekutz AC (1997) Activation of endothelial cells via antibody-enhanced dengue virus infection of peripheral blood monocytes. J Virol 71: 4226–4232. 9151809 10.1128/jvi.71.6.4226-4232.1997 PMC191637

[40] Schlesinger JJ, Foltzer M, Chapman S (1993) The Fc portion of antibody to yellow fever virus NS1 is a determinant of protection against YF encephalitis in mice. Virology 192: 132–141. 8517015 10.1006/viro.1993.1015

[41] Lin CW, Liu KT, Huang HD, Chen WJ (2008) Protective immunity of *E. coli*-synthesized NS1 protein of Japanese encephalitis virus. Biotechnol Lett 30: 205–214. 17876533 10.1007/s10529-007-9529-9

[42] Chung KM, Nybakken GE, Thompson BS, Engle MJ, Marri A, et al (2006) Antibodies against West Nile Virus nonstructural protein NS1 prevent lethal infection through Fc gamma receptor-dependent and -independent mechanisms. J Virol 80: 1340–1351. 16415011 10.1128/JVI.80.3.1340-1351.2006 PMC1346945

[43] Diamond MS, Pierson TC, Fremont DH (2008) The structural immunology of antibody protection against West Nile virus. Immunol Rev 225: 212–225. 18837784 10.1111/j.1600-065X.2008.00676.x PMC2646609

[44] Falgout B, Bray M, Schlesinger JJ, Lai CJ (1990) Immunization of mice with recombinant vaccinia virus expressing authentic dengue virus nonstructural protein NS1 protects against lethal dengue virus encephalitis. J Virol 64: 4356–4363. 2143542 10.1128/jvi.64.9.4356-4363.1990 PMC247903

[45] Chung KM, Thompson BS, Fremont DH, Diamond MS (2007) Antibody recognition of cell surface-associated NS1 triggers Fc-gamma receptor-mediated phagocytosis and clearance of West Nile Virus-infected cells. J Virol 81: 9551–9555. 17582005 10.1128/JVI.00879-07 PMC1951387

[46] Cheng HJ, Lei HY, Lin CF, Luo YH, Wan SW, et al (2009) Anti-dengue virus nonstructural protein 1 antibodies recognize protein disulfide isomerase on platelets and inhibit platelet aggregation. Mol Immunol 47: 398–406. 19822367 10.1016/j.molimm.2009.08.033

[47] Heinz FX, Stiasny K (2012) Flaviviruses and flavivirus vaccines. Vaccine 30: 4301–4306. 22682286 10.1016/j.vaccine.2011.09.114

[48] Swaminathan S, Batra G, Khanna N (2010) Dengue vaccines: state of the art. Expert Opin Ther Pat 20: 819–835. 20384534 10.1517/13543771003767476

[49] Coller BA, Clements DE (2011) Dengue vaccines: progress and challenges. Curr Opin Immunol 23: 391–398. 21514129 10.1016/j.coi.2011.03.005

[50] Murrell S, Wu SC, Butler M (2011) Review of dengue virus and the development of a vaccine. Biotechnol Adv 29: 239–247. 21146601 10.1016/j.biotechadv.2010.11.008

[51] Thomas SJ, Endy TP (2011) Critical issues in dengue vaccine development. Curr Opin Infect Dis 24: 442–450. 21799408 10.1097/QCO.0b013e32834a1b0b

[52] Perng GC, Lei HY, Lin YS, Chokephaibulkit K (2011) Dengue vaccines: challenge and confrontation. World J Vaccines 1: 109–130.

[53] Sabchareon A, Wallace D, Sirivichayakul C, Limkittikul K, Chanthavanich P, et al (2012) Protective efficacy of the recombinant, live-attenuated, CYD tetravalent dengue vaccine in Thai schoolchildren: a randomised, controlled phase 2b trial. Lancet 380: 1559–1567. 22975340 10.1016/S0140-6736(12)61428-7

[54] Teoh EP, Kukkaro P, Teo EW, Lim APC, Tan TT, et al (2012) The structural basis for serotype-specific neutralization of dengue virus by a human antibody. Sci Transl Med 4: 139ra83. 10.1126/scitranslmed.3003888 22723463

[55] Li PC, Liao MY, Cheng PC, Liang JJ, Liu IJ, et al (2012) Development of a humanized antibody with high therapeutic potential against dengue virus type 2. PLoS Negl Trop Dis 6: e1636. 22563515 10.1371/journal.pntd.0001636 PMC3341331

[56] Henriques HR, Rampazo EV, Gonçalves AJS, Vicentin ECM, Amorim JH, et al (2013) Targeting the non-structural protein 1 from dengue virus to a dendritic cell population confers protective immunity to lethal virus challenge. PLoS Negl Trop Dis 7: e2330. 23875054 10.1371/journal.pntd.0002330 PMC3715404

