# Supplementary figures and images for: Protection against Dengue Virus Infection in Mice by Administration of Antibodies against Modified Nonstructural Protein 1
Source: PLoS One. 2014 Mar 21;9(3):e92495. doi: 10.1371/journal.pone.0092495 (PMC3962419; doi:10.1371/journal.pone.0092495)

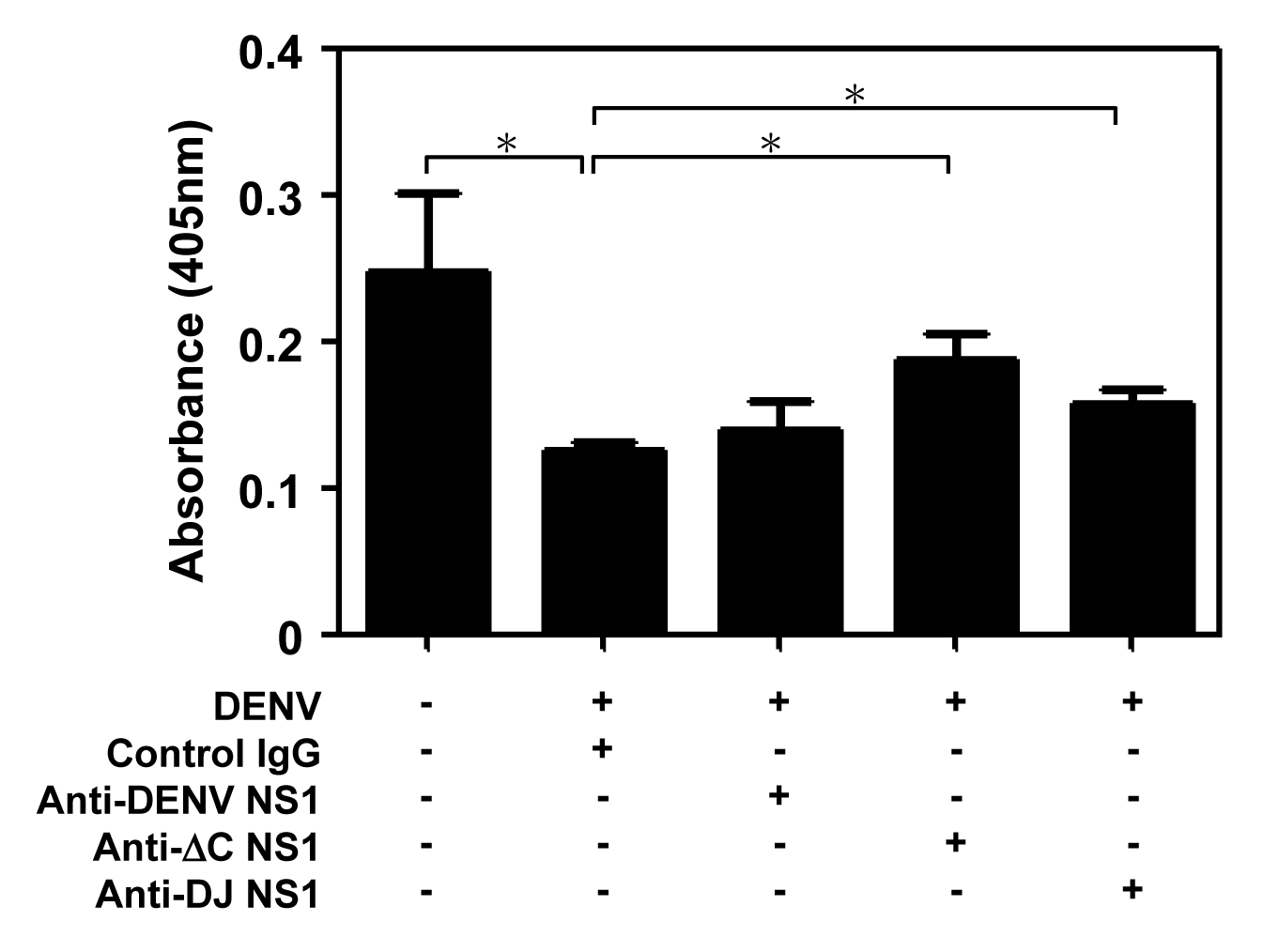

Supplement: Figure S1 — Anti-ΔC NS1 and anti-DJ NS1 Abs rescue DENV-induced thrombin activity inhibition. Thrombin activity was detected by adding chromogenic substrates S-2238 to the mouse plasma and incubating at room temperature for 1 h. The optical density value at 405 nm was measured every 10 min with a VersaMax microplate reader [34]. The inhibition of thrombin activity occurred in the DENV plus control IgG group compared to medium control and was partially rescued by anti-ΔC NS1 and anti-DJ NS1 Abs treatment (n = 4/group). * P<0.05. (TIF) [file pone.0092495.s001.tif]

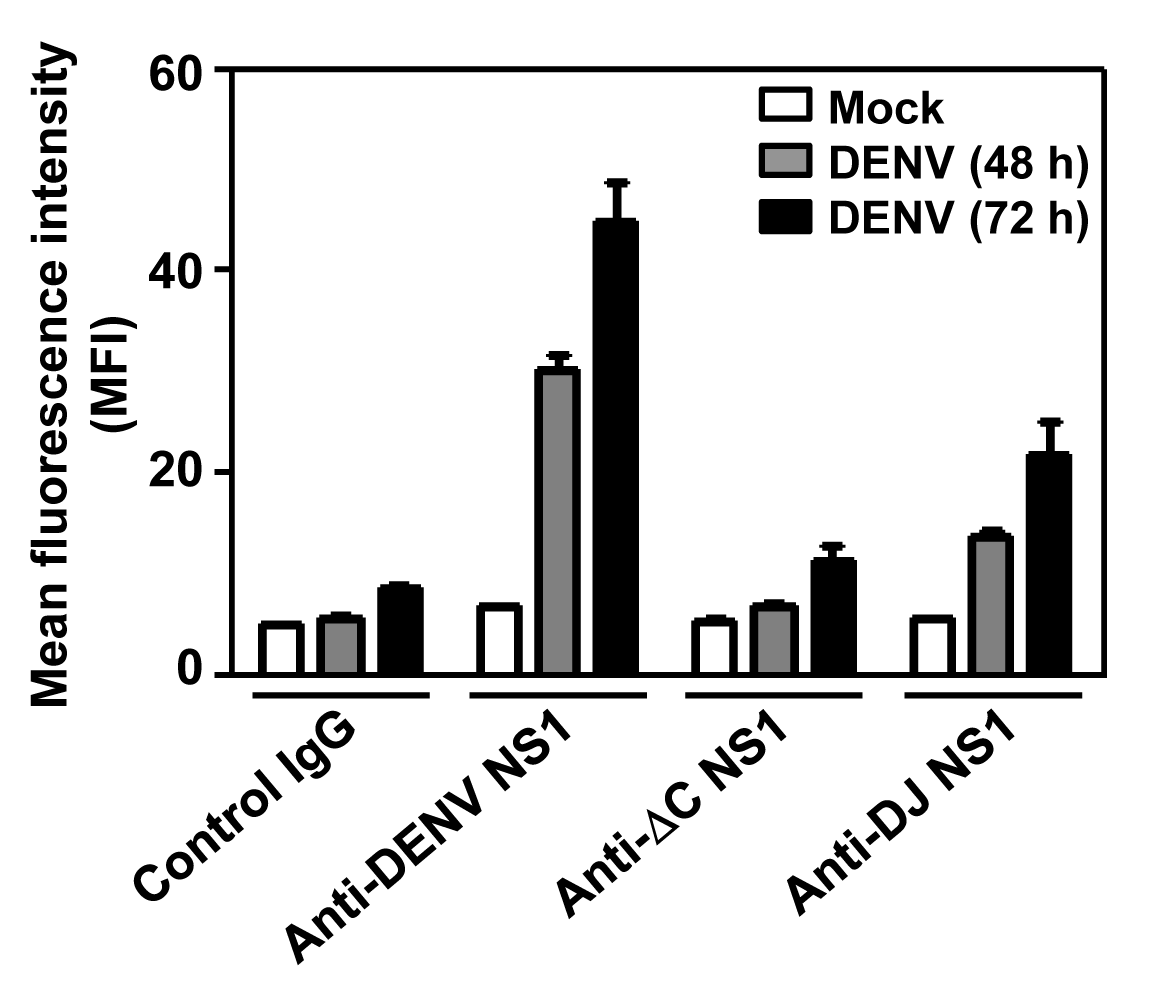

Supplement: Figure S2 — Anti-ΔC NS1 and anti-DJ NS1 Abs possess binding activity to DENV-infected endothelial cells. HMEC-1 cells were infected with DENV (MOI = 10) for 48 or 72 h. Non-fixed cells were stained with control IgG, anti-DENV NS1, anti-ΔC NS1 or anti-DJ NS1 Abs (2.5 μg), followed by Alexa 488-conjugated donkey anti-mouse IgG staining and analyzed by flow cytometry. The averages ± SD obtained from triplicate cultures are shown. (TIF) [file pone.0092495.s002.tif]

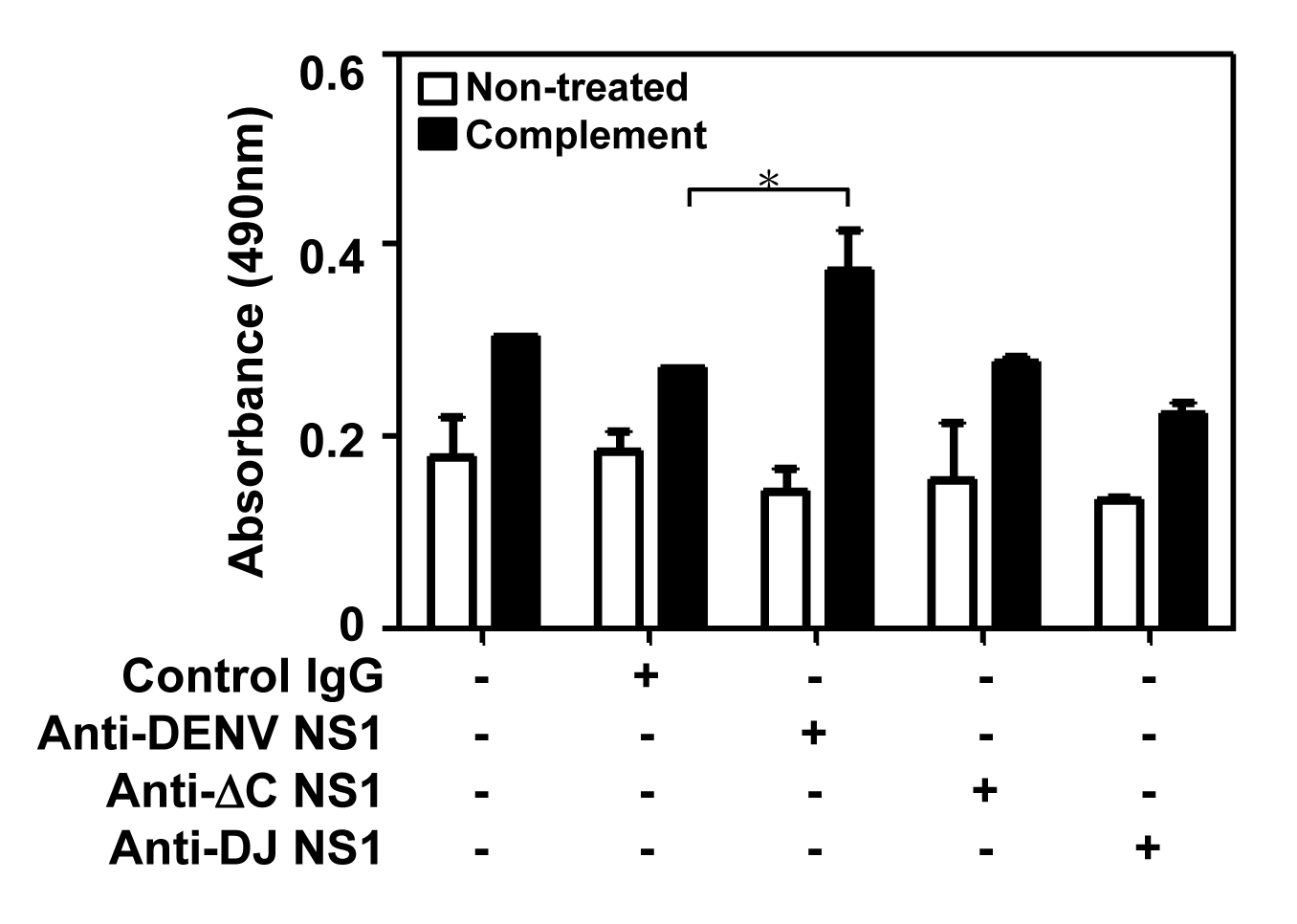

Supplement: Figure S3 — Anti-DENV NS1 Abs cause complement-mediated cytolysis in uninfected cells. Uninfected HMEC-1 cells were incubated with control IgG, anti-DENV NS1, anti-ΔC NS1 or anti-DJ NS1 Abs (50 μg/ml) for 1 h at 4°C and then incubated with or without complement for 4 h at 37°C. Cell culture supernatants were collected and detected for the release of lactate dehydrogenase (LDH). The averages ± SD obtained from triplicate cultures are shown. * P<0.05. (TIF) [file pone.0092495.s003.tif]
